# 

**Published:** 1799-09

**Authors:** 


### NEW MEDICAL PUBLICATIONS IN AUGUST.

Lectures on Diet and Regimen: being a Syftematic Inquiry into the moft rational means of preferving Health, and prolonging Life; together with phyfiological and chemical explanations, calculated chiefly for the ufe of families, in order to banifh the prevailing abufes and prejudices in Medicine. *The fecond edition, improved and enlarged, with confiderable additions.* By A. F. M. Willich, m. d. 8vo. 708 pages. Price 9s. in boards, or 10s. 6d. on wove paper. *Longman* and *Rees.*

The Natural Hiftory of the Tea-tree; with obfervations on the medical qualities of Tea, and on the effects of Tea-drinking; with coloured Plates. *A new edition:* By J. C. Lettsom, m. d. 4to. 102 pages. 10s. 6d. *Dilly.*

Obfervations deduced from Facts and Experiments, tending to evince the non-exiftence of Typhus-contagion, interfperfed with remarks on Animal Life, and on thofe laws by which it is governed; alfo with fome remarks on the nature of thofe difeafes which are epidemic at fea. By J. Franks, Surgeon in his Majefty's navy, &c. 8vo. 70 pp. *Johnfon.*

A Treatife on Bilious Difeafes and Indigeftion; with the effects of Quaffy and Natron in thofe diforders: By J. Gibson, m. d. Surgeon in the Royal Navy, &c. 8vo. 68 pp. 2s. *Murray* and *Highley.*

An entire new Treatife on Leeches, wherein the nature, properties, and ufes of that fingular and valuable animal are clearly explained. By J. Horn, Apothecary, &c. 8vo. 30 pp. 1s. 6d. *Symonds.*

Obfervations on the difeafed and contracted urinary bladder, and frequent painful micturition, with fome cautions refpecting the ufe of the cauftic bougie in the treatment of ftrictures in the urethra: including a paper on the fcirrho-contracted rectum, to which the Medical Society of London adjudged a prize medal in the year 1798. By John Sherwen, m. d. Member of the Corporations of Surgeons. Price 1s. 6d. *Johnfon,* and *Murray* and *Highly.*

---

### NEW MEDICAL PUBLICATIONS IN GERMANY.

*Sammlung auferlefener Abhandlungen,* &c.—A Collection of Treatifes, felected for the ufe of Medical Practitioners. 8vo. Vol. XVIII. *Leipzig.* Dyk.

*Kurze Darftellung,* &c.—A concife view of the chemical inquiries into the nature of the different gafes. By Dr. A. N. Scherer. 8vo. *Weimar.* Gædicke.

*Verfuch einer Theorie der elektrifchen Erfcheinungen.*—An Effay towards a theory of the phenomena occurring in Electricity: By L. A. Von Arnim. 8vo. 146 pp. with a Plate. *Halle.* Gebauer.

*Der*

*Der Arzt für Freudenmædchen*, &c.—The Medical Guide for the Votaries of Pleasure, &c. 8vo. 138 pp. Bremen.

*Ueber das Aufziehen fremder Kœrper*, &c. On the extraction of foreign bodies from the œsophagus and the trachea: By T. G. ECOLDT. 4to. with five plates. *Leipzig*; Tauchnitz.

*Analytische Tabellen*, &c.—Analytical Tables, exhibiting the different species of Minerals; being an Attempt towards a more correct Method of determining and recognizing them, without a Teacher. By A. J. G. B. BATSCH, Prof. at Jena: with a Plate (28 grosch. or about 3s. 6d.) *Jena*; Gœpferdt.

*Handbuch der praktischen Heilmittellehre*, &c.—A Manual of practical Therapeutics, for the use of the young Practitioner, as well as the Friend of the Veterinary Art. Vol. I. containing the Therapeutics of external diseases. 8vo. (12 grosch. or about 2s.) *Leipzig*; Seeger.

*Apothekerlexicon*:—A Pharmaceutical Dictionary. By SAM. HAHNEMANN. 8vo. two vols. *Leipzig*; Crusius.

G. F. HOFFMANNI, *Plantæ lichenosæ delineatæ et descriptæ*. Vol. VIII. *Fasc. 3tius cum figuris coloratis. Folio.* (3 rix-doll. 12 grosch.) or about 13s. 6d.) *Ibid.*

---

NEW MEDICAL PUBLICATIONS IN FRANCE.

*Experiences sur le Galvanisme*, &c.—Experiments on Galvanism, and likewise on the irritation of the muscular and nervous fibres: By F. A. HUMBOLDT, translated from the German; with Additions, by J. F. A. JADELOT. 8vo. 600. pp. (Price 6 livres) *Paris*; Fuchs.

*Observations sur l'Operation dite Césarienne*, &c.—Observations on a successful instance of the Cæsarean operation: to which is added a Description of a new method of performing it. By Cit. J. A. MILLOT, Accoucheur. 8vo. 38. pp. *Paris*; Croullebois.

---

### TO CORRESPONDENTS.

*We have already apprized our Correspondents, that we cannot, consistently with our plan, admit anonymous strictures on any communications which have been published in this Journal, with the signatures of their authors. We are confident that many of our respectable friends have been induced to honour us with their contributions, in consequence of the conviction, that they were perfectly secure from the shafts of illiberal or anonymous criticism.*

The letter dated August the 12th, and signed "Observator," contains such assertions relative to the Inoculation of the Cow-pox, as cannot be published without either proof or authority. If the 'Observator' can procure the former, and authenticate it with the signature of his *real* name, we shall not hesitate a moment to comply with his wishes.

Another paper signed J. B. August 17th, must be delayed for similar reasons.

The letter with the signature D. W. on the Prevention and Cure of Dysentery, arrived too late for insertion in the present Number.

The interesting Account of an extra-uterine Fœtus, transmitted to us by a Medical Friend at Norwich, shall certainly appear in our next.

Mr. L——'s paper on the Aqua Ammoniæ Acetata must be likewise postponed for want of room.

